# Biomarkers for early stage of acute respiratory distress syndrome in septic patients: surfactant protein D and Clara cell protein

**DOI:** 10.1186/cc12038

**Published:** 2013-03-19

**Authors:** A Kuzovlev, V Moroz, A Goloubev, S Polovnikov

**Affiliations:** 1 V.A. Negovsky Scientific Research Insitute of General Reanimatology RAMS, Moscow, Russia; 2 N.N. Burdenko Main Military Hospital, Moscow, Russia

## Introduction

The injury of alveolar epithelium and endothelium is the basis of pathogenesis for the early stage of acute respiratory distress syndrome (ARDS). A prompt detection of this injury will provide us with a possibility for early ARDS diagnosis and treatment. Currently no biomarkers of alveolar epithelial injury are clinically available. The purpose of our study was to investigate the role of surfactant protein D (SPD) and Clara cell protein (CCP) as biomarkers of early ARDS.

## Methods

This observational study in ICU mechanically ventilated septic patients with intra-abdominal surgical infections was carried out at the V.A. Negovsky Research Institute of General Reanimatology in 2010 to 2012. ARDS was diagnosed and staged according to the research institute criteria [1] and the American-European criteria. Plasma concentrations of SPD and CCP were measured on ARDS diagnosis (day 0) and days 3, 5, and 7 by immunoenzyme essay (Bio Vendor, USA). Data were statistically analyzed by STATISTICA 7.0, ANOVA method, and presented as mean ± σ, ng/ml. *P <*0.05 was considered statistically significant. Areas under the receiver operating curves (ROC) were calculated.

## Results

Fifty-five patients (out of 250 screened) were enrolled according to the inclusion/exclusion criteria. Patients were assigned into groups: ARDS (*n *= 30; subgroups - ARDS stage 1 (*n *= 15), stage 2 (*n *= 15)) and noARDS (*n *= 25). In the ARDS group SPD was higher at all points than in the noARDS group (day 0 - 352.5 ± 287.8 vs. 141.0 ± 103.4; day 3 - 278.6 ± 204.8 vs. 151.8 ± 125.9; day 5 - 339.9 ± 331.4 vs. 162.3 ± 138.8; day 7 - 339.9 ± 300.1 vs. 169.8 ± 154.5; *P <*0.05). SPD levels were lower at ARDS stage 1 in comparison with stage 2 (day 0 - 154.6 ± 125.2 vs. 451.8 ± 299.2; day 3 - 129.3 ± 74.9 vs. 362.9 ± 202.0; day 5 - 167.8 ± 120.4 vs. 428.5 ± 372.4; day 7 - 186.2 ± 127.1 vs. 415.5 ± 337.0; *P <*0.05). Plasma SPD had a good diagnostic capacity for stage 1 ARDS: SPD on day 0 ≤253.0 ng/ml yielded a sensitivity of 90% and specificity of 74% (AUC = 0.83; 95% CI = 0.631 to 0.951; *P *= 0.0001). No such differences for CCP were detected.

## Conclusion

Plasma SPD level ≤253.0 ng/ml is a sensitive and specific biomarker of the early stage of ARDS in septic patients.
